# A Neglected Zoonosis in Albania: why Echinococcosis is Becoming a Surgeon’s Exclusivity?

**DOI:** 10.4084/MJHID.2014.013

**Published:** 2014-02-16

**Authors:** Arben Pilaca, Gentian Vyshka, Arben Pepa, Kastriot Shytaj, Valentin Shtjefni, Arben Boçari, Arben Beqiri, Dhimitër Kraja

**Affiliations:** 1 Service of Infective Diseases, University Hospital Centre “Mother Theresa”, Tirana, Albania; 2 Biomedical and Experimental Department, Faculty of Medicine, University of Tirana, Albania; 3 Obstetrical and Gynecological Hospital, Tirana, Albania; 4 Faculty of Medical and Technical Sciences, University of Medicine in Tirana, Albania; 5 Institute of Veterinary Care, Tirana, Albania; 6 Faculty of Veterinary Medicine, Agricultural University of Tirana, Albania; 7 Service of Surgery, University Hospital Centre “Mother Theresa”, Tirana, Albania

## Abstract

Echinococcosis is an endemic zoonosis in the Mediterranean area, with Albania interested actually to a level that is becoming a public health concern. Authors describe preliminary data from the only tertiary (university) medical facility of Albania, positioned in the capital of the country (Tirana), with 333 new cases diagnosed and treated during the period 2005 – 2011. Out of all these 333 new cases an impressive majority of 91% had a surgical treatment right from the first admission, rendering the disease almost a surgical exclusivity. Even more, 80% of all patients from the study group were hospitalized straightforwardly in surgical wards, with options of surgical intervention’s percentages outrunning figures from other sources and authors of the same geographical area. Such a situation, together with a very important level of patients’ origin from highly urbanized areas such as those of the capital, suggest the necessity of well-organized interventions, among which might be the mandatory notification of all human cases with Echinococcus infection.

## Introduction

Echinococcosis is a chronic and zoonotic infection, actually representing one of the three major helminth diseases that cause a major public health concern especially in developed countries, together with cysticercosis and fascioliasis.^1^

Echinococcosis is synonymously known as the hydatid disease, with the term ‘hydatid’ deriving from the Greek ‘*hyd r’*, meaning water, and thus reflecting the cystic character of the infective process. Controversial data regarding the epidemiology and the severity of the infection among humans are available, with some authors offering an optimistic perspective of a dramatic fall in the incidence and prevalence of the most common form of the disease, namely the cystic echinococcosis.^2^ However, and in spite of large preventive and therapeutic interventions, cystic echinococcosis remains a frequent condition in developed and undeveloped countries.^3^,^4^

Classic cystic echinococcosis (CE) is caused by *E. granulosus*, one of the two major species of the genus Echinococcus able to infect humans. *E. multilocularis* is the second most important pathological species of this genus. These species are respectively responsible for CE, and for the alveolar echinococcosis (AE). AE is caused from *E. multilocularis* and represents the other clinical picture related to these tapeworms, able to infest humans as well as other mammalian intermediate hosts. Wildlife animals and domesticated pets might be infested in a variety of ways or forms, and from different species of Echinococcus.^5^,^6^ Thus, *E. granulosus, E. multilocularis, E. vogeli* and *E. oligarthus* have shown to have different but intrinsic pathological potential regarding the ability to infest humans, with other species of the same genus having only un unclear infective potential, if at all.^7^,^8^

Multiple studies have scrutinized risk factors and environmental changes that influence on the global spread of the infection. Host characteristics as well as host population dynamics and density are among the most studied in a variety of animals. Obviously canids, dogs in first line, serve as definitive host; foxes, jackals, dingo, hyena, wolves and raccoon-dogs might be definitive hosts as well.^8^,^9^ The list of intermediate hosts might be even longer, with rodents on the top, and with a variety of domesticated animals (sheep, pigs, goats) serving that role, but without excluding even exotic or at risk of extinction wildlife organisms, such as hippopotamus, giraffes, antelopes (kudu) and so on.^8^,^10^,^11^,^12^

Echinococcosis is considered as an endemic zoonotic disease in the Mediterranean area.^2^ Some authors suggest, with obvious and sound reasons, that this disease with variegate clinical features, is posing a severe threat even to the public health level.^13^ Both major clinical forms, CE and AE, have been found present in different Mediterranean countries, although CE and *E. granulosus*, having the latter an intrinsic affinity for warmer climates, represent the overwhelming form. Much more disturbing is the fact that that *E. multilocularis*, the causative factor of AE, generally considered as a species occurring only sporadically in this geographical area, is however recently found to expand his habitat continuously.^13^,^14^

Although representing a major public health problem and a considerable burden of disease for several Mediterranean countries, epidemiological data are scarce, and the precise incidence among humans is unknown for CE as well as for AE in Albania and in countries neighboring it.^15^ With a worldwide prevalence of approximately six million, CE needs global approaches and strategies to prevent further progression.^3^ Among the factors explaining this persistence of the disease, sources quote the climate changes and the warmer temperatures.^16^,^17^ Host density is another major factor; if there are no epidemiological data regarding human incidence of echinococcosis in Albania, no data either are offered regarding stray dog populations in our country.

The aim of the present study was to systematize preliminary data gathered from our University Hospital Facility in Tirana, regarding cases of echinococcosis diagnosed and treated during recent years (2005–2011). An insight to epidemiological characteristics of this zoonosis in Albania is given as well, and a geographical distribution of cases was made, aiming at localizing districts of the country being at “high risk” for possible outbreaks, due to the actual high expression of the disease.

## Materials and Methods

This retrospective and single-centre study was performed in the only University Hospital Centre of Tirana, capital of Albania. Due to panoply of reasons, district hospitals almost constantly refer cases to this facility; however the data are not meant to represent the overall country presence of the disease, since many cases of Echinococcosis might go untreated.

UHC of Tirana is organized in several services that function as clinical wards; all data and medical files are deposited in a central statistical office, with medical doctors and authorized personnel able to access their content decades after the discharge of a patient.

This is a descriptive study of the hospitalizations at the University Hospital Centre “Mother Theresa” of Tirana (UHC of Tirana), during the period 2005 – 2011, regarding all cases whose admission diagnosis was ‘*echinococcosis*’.

A study of the cases was made after carefully controlling the central statistical office of UHC of Tirana, taking note of following details:

1. Hospitalization period (days of admission).
2. Age of patients.
3. Overall distribution in different clinical services of UHC of Tirana.
4. Time distribution (total yearly figures from 2005 to 2011).
5. Clinical localization of the disease (visceral distribution; main focus upon admission).
6. Geographical origin of the admitted patient.
7. Size of cysts.

## Results

During the period 1^st^ January 2005 – 31^st^ December 2011 we had a total of 385 hospitalizations with the admission diagnosis of ‘Echinococcosis’, for a total of 333 patients (de novo and recurrent admissions), with *303 cases operated*. Only one case had a fatal outcome, and is not included in the database of the Table 2.

According to our data, 91% of the cases of echinococcosis admitted in the UHC of Tirana during a seven years period, was initially admitted or referred during the same hospitalization at the UHC, in a surgical facility, and underwent a surgical intervention.

The mean period of *hospitalization* is given in the Table 1, where are considered the services of UHC accounting for the majority of all cases admitted during this period.

The *mean age* of patients forming the study group (a total of 333 patients) resulted 36 years old (from a minimum of six month, to a maximum age of 77 years).

Overall distribution of cases in the *different clinical services* of UHC of Tirana is described at the Table 2.

Time distribution (total yearly figures from 2005 to 2011) is described in the Table 3. Worthwhile is stating that it has been an almost constant figure year after year (2005 – 2011) with a minimum of 44 cases during the year 2010 and a maximum of 63 cases (2007, 2009), thus the yearly level of newly diagnosed cases did not changed substantially.

The clinical localization of the disease (visceral distribution; in terms of the main focus upon admission) is described in the Table 4. The distribution of cases is made in separate age ranges (decennia).

**Figure 1:**
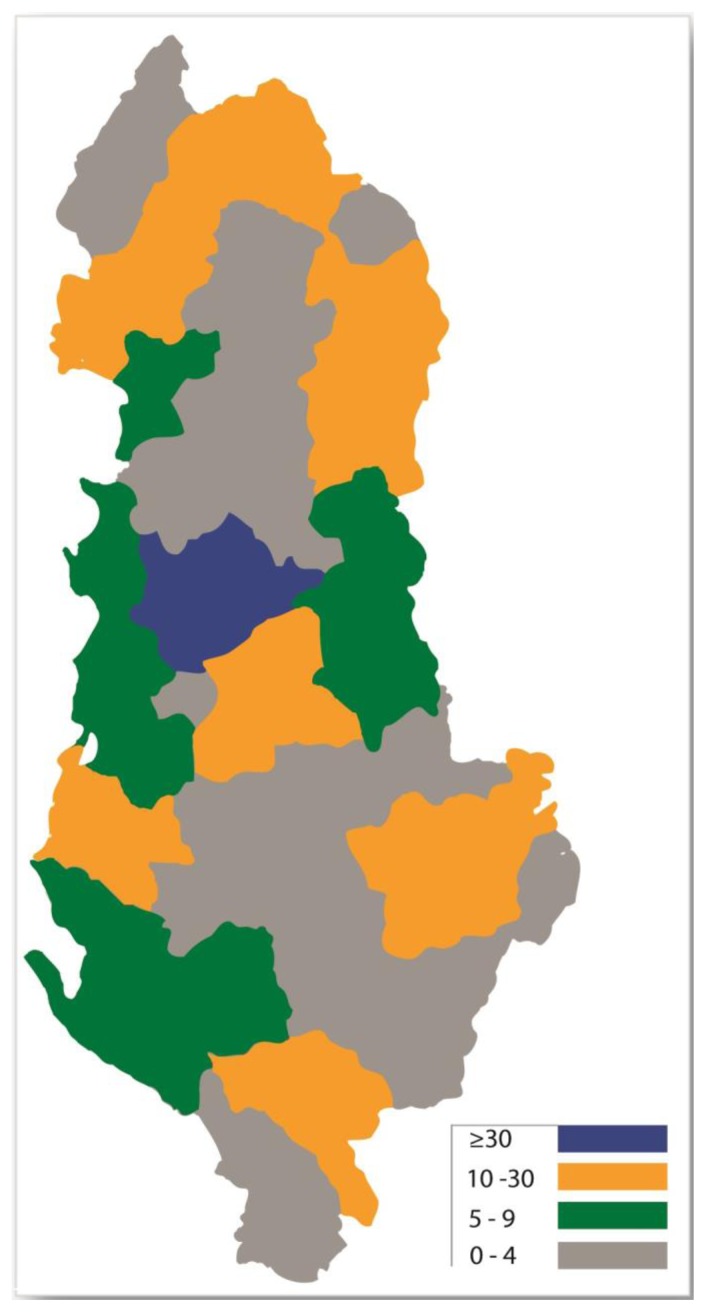
Illustrative map of the geographical distribution of cases according to different administrative districts of Albania. Different colors present the absolute distribution of patients hospitalized at the UHC of Tirana, during the period 2005–2011. (For explanations see the text below).

The geographical distribution of the cases was made accordingly with the administrative map of Albania, shown in the Figure 1. In order to simplify the exposition of data, we have separated graphically the districts of the country in the map below through illustrating the number of cases with a different color.

Thus, in the map below (Figure 1) we have separated different district of the country through using following illustrative colors:

*Blue*; for administrative districts having more than thirty cases hospitalized during the study period;
*Yellow-orange*; for districts having 10–30 cases hospitalized during the same period;
*Green*; for districts having 5–9 hospitalizations in total (2005–2011);
*Grey*; for districts having less than five cases in total for the study period.

According to the data we gathered, we had a total of 99 patients hospitalized during the study period, originating from the district of Tirana (capital of the country, and surrounding areas). Thus, this city and the respective district are shown in the map with *blue* color. In fact, the absolute number of cases coming from this district was overwhelmingly higher when compared with other districts of the country.

With *yellow-orange* color there are shown a total of eight districts of Albania, including four northern administrative districts (namely Kukës [with 30 patients], Dibra [17 patients], Shkodra [13 patients] and Tropoja [10 patients]). With the same color we illustrated four other districts almost symmetrically positioned in the lower half of the country’s map, including a central Albanian district (Elbasan with 10 patients); a south-west district close to the Adriatic seashore (Fier with 13 patients); a south-east district neighboring Greece (Korça with 21 patients) and a southern district almost at the lower extreme of the map (Gjirokastra with 14 patients).

Other administrative districts of the country having less than 10 hospitalizations during the entire study period were depicted in *green* and *grey* (see map). The distribution of the cases in these districts followed a disparate order as well, although districts close to the Adriatic seashore had somehow higher absolute figures (port cities of Durrës and Vlora [shown in green] with 9 patients each); when compared to southern – southeastern districts neighboring Greece, with lower absolute figures (Devoll, Delvina and Skrapar [shown in grey] each of them with 1 patient for the entire study period).

The size of cysts was registered only in a minority of our cases (114 patients), and the available data were collected from echography findings. Due to this gap we cannot refer exhaustive values of cyst sizes in our study group; however the size of the cysts’ diameter varied from a minimum of 2 centimeters to a maximum of 15 centimeters, in this subgroup of patients where the cyst size was registered.

## Discussion

Echinococcosis (hydatidosis) is an endemic disease for the Mediterranean area, and Albania will obviously be interested to the same extent as other countries of the region. Since no exhaustive or thorough statistical and epidemiological study is available, comparing the epidemiological situation with that of neighboring countries still remains a challenge.

As far as it regards a non-reportable infection, collecting statistics for an entire country might be virtually impossible. Nevertheless, attempts to profile and to analyze echinococcosis in endemic regions have been made.^18^ Another non trivial difficulty is related to the panoply of clinical features through which echinococcosis might present, becoming in certain situation a diagnostic conundrum. Our study confirms as well an almost universal finding, that the majority of cases of echinococcosis are confined to the liver and to the lungs.

Hydatid cyst will show a clear mass effect and therefore will be a surgical exclusivity in many settings. Highly peculiar clinical forms are reported, such as Budd-Chiari syndrome, left ventricle hydatid cyst mimicking heart attack, genital localization with scrotal extension and so on.^19^,^20^,^21^ Such a variety of localizations is related to the fact that hydatidosis affects almost all body regions.

In the present paper we have encountered two worth mentioning phenomena:

- A very high proportion of patients (99 from the total study group of 333) originating from the district of Tirana, comprising the capital and its suburban areas;
- An excessively high proportion of surgical approach as well, with 91% of all patients forming our study group that were operated during the initial admission at the UHC of Tirana, and even with almost 80% of all patients admitted straightforward in a surgical facility (here including general, special, pediatric and neurological surgery services).

The fact that we had the majority of cases originating from Tirana might be artifactual, since UHC itself is situated in this capital city, and inhabitants of the district will have an easier access to the facility. However, UHC of Tirana is the single tertiary (university) hospital in Albania; therefore all difficult and complicated cases will be referred herein. This is much true nowadays when almost all specialized medical help, staff and equipment, is focused inside this centre, with periphery and remote districts suffering from inexistent or incompetent medical services.

The high proportion of cases originating from Tirana might be also related to other reasons. High and aggressive urbanization of the capital and its suburban areas, with the sanitary problems that such a process with entail, here including even the garbage disposal and landfills (Sharra landfill among other) in the vicinity of the city, have been at the focus of media reports.^22^ The presence of domesticated animals that are part of the transmitting chain of this infection to humans, such as sheep and dogs, inside landfills and in the city itself has been a constant concern. Serious attempts have been made to study the intestinal parasite fauna of dogs of the suburban area of Tirana, with Echinococcus being part of the list.^23^

The other major suggestion of the present study was the fact that actually in Albania, and in the only tertiary medical facility of the country (UHC), echinococcosis has practically become a surgical exclusivity. Approximately 90% of the entire study group admitted with this diagnosis during 2005–2011 ended up with a surgical intervention. But probably the most important fact is that 80% of all patients were right from the start hospitalized in a surgical ward, thus the condition has (almost) never been consulted from infectious disease specialists, with treatment focused alone in the surgery. In fact, from all patients treated surgically, only 23 of them had later (within six months from the intervention) an infectionist consultancy, and none of these patients were receiving an ad hoc pharmacological therapy prior to surgery.

This very high level of surgical interventions contrasts with findings from other tertiary centers, with authors from countries neighboring Albania referring surgery as a single option only in 10% of the study group.^4^

Under these circumstances, and in view of the endemic situation in the Mediterranean area, mandatory notification of all human cases of Echinococcus infection, through taking necessary steps toward rendering the condition obligatorily a reportable one, seems more than logical.

## Figures and Tables

**Table 1:** Mean hospitalization period (days)

*Respective service (clinic) of UHC of Tirana*	*Percentage over the entire study group (384 hospitalization in total, de novo plus recurrences)*	*Average staying (days)*
Gastro-hepatological service	*13.5*	8.83
1^st^ clinic of surgery	*55.5*	6.09
2^nd^ clinic of surgery	*19.0*	9.43
Pediatric surgery	*2.6*	13.5

**Table 2:** Distribution of cases according to the service of UHC where initially hospitalized*

*Service of UHC where first diagnosis (hospitalization) took place*	*Total number of patients*	*Percentage over the entire study group (384 hospitalization in total, de novo plus recurrences)*
Allergology	1	0.3
Gastro-hepatology	52	13.5
Hematology	1	0.3
Hypertonic disease service	2	0.5
Infectious diseases service	14	3.6
Cardiac surgery	3	0.8
***Special surgery (2****^nd^* ***clinic)***	***73***	***19.0***
***General surgery (1****^st^* ***clinic)***	***213***	***55.5***
***Neurosurgery***	***6***	***1.6***
Neurology	1	0.3
***Pediatric surgery***	***10***	***2.6***
Central ICU (intensive care)	8	2.0

**Table 3:** Distribution of cases on a yearly basis

Year	Total no. of patients diagnosed during that year	Percentage (relative to the entire study period)
2005	48	12.5 [%]
2006	61	15.9
2007	63	16.4
2008	58	15.1
2009	63	16.4
2010	44	11.5
2011	47	12.2

**Table 4:** Localization of main infectious focus upon admission

Age range (years)	Biliary tree	Genitals	Liver	Spleen	Myocardium	Lung	Kidney	Vertebra	Other
1–5	0	0	4	0	0	2	0	0	0
6–15	0	0	34	0	0	30	0	0	9
16–25	0	0	37	0	1	1	0	0	3
26–35	0	0	43	0	0	1	0	0	2
36–45	0	0	32	1	1	0	0	1	2
46–55	1	1	32	0	0	3	0	1	3
56–65	0	0	46	0	2	0	1	1	6
>65 yrs.	0	0	27	0	0	0	1	1	3
**Total**	**1**	**1**	**255**	**1**	**4**	**37**	**2**	**4**	**28**
